# A Nursery-Based Cooking Skills Programme with Parents and Children Reduced Food Fussiness and Increased Willingness to Try Vegetables: A Quasi-Experimental Study

**DOI:** 10.3390/nu12092623

**Published:** 2020-08-28

**Authors:** Ada L. Garcia, Emma Brown, Tom Goodale, Mairi McLachlan, Alison Parrett

**Affiliations:** 1 Human Nutrition, School of Medicine, Dentistry and Nursing, College of Medical, Veterinary and Life Sciences, University of Glasgow, Glasgow G31 2ER, UK; Emma.Brown@lcfhp.co.uk (E.B.); Alison.Parrett@glasgow.ac.uk (A.P.); 2 Lanarkshire Community Food and Health Partnership, Bargeddie G69 7TU, UK; Mairi.McLachlan@lcfhp.co.uk; 3 Academic Achievement Team, Library Services, Liverpool John Moores University, Liverpool L3 5UX, UK; t.r.goodale@ljmu.ac.uk

**Keywords:** preschool child, vegetables, eating behaviour, cooking skills, parent, repeated exposure, sensory learning

## Abstract

Children’s fussy eating is associated with a reduced vegetable intake. This quasi-experimental study evaluated “Big Chef Little Chef” (BCLC), a nursery-based cooking skills programme aimed at reducing food fussiness and increasing willingness to try green vegetables by incorporating repeated exposure and sensory learning. Parent and child (3–5 years) dyads attended BCLC for four/1.5 h weekly sessions. A comparison group was recruited after BCLC completion and attended a single education session at week 1. A questionnaire measured food fussiness at week 1 and week 4. At week 4, all children were offered six green vegetables (raw and cooked) and an average score (1 = did not try; 2 = tried it/ate some; 3 = ate it all) was calculated for willingness to try vegetables. In total, 121 dyads (intervention: *n* = 64; comparison: *n* = 57) participated. The food fussiness score (1 min–5 max) in the intervention group decreased significantly from 3.0 to 2.6 (*p* < 0.01) between time points, while there was no change in the comparison group (3.1 (week 1) and 3.0 (week 4)). The intervention group was more willing to try green vegetables with significantly higher (*p* < 0.001) median scores for raw and cooked vegetables (2.5 for both) compared with the comparison group (2.0 and 1.7, respectively). The BCLC reduced food fussiness and increased willingness to try green vegetables.

## 1. Introduction

Fussy eating, also referred to as picky, faddy or choosy eating, in children can involve the rejection of both familiar and unfamiliar food items [1]. This can result in the provision of different meals or foods for the child compared with the rest of the family [2]. Fussy eating is associated with reduced diet variety including reduced intakes of fruits and vegetables (F and V) [3,4,5], wholegrains [5] and meat and fish [4,5]. In the UK, 12%–15% of parents in the Avon Longitudinal Study of Parents and Children (ALSPAC) cohort described their child (aged 38–65 months) as “very choosy” in regard to food, while 40%–41% described them as “quite choosy” [6]. This is important as dietary patterns formed during childhood may continue into adolescence and adulthood. Children in the ALSPAC cohort that were described as “very choosy” with food at 3 years old were found to consume significantly less F and V at both 10 and 13 years compared with those who were not identified as choosy with food [7]. Low F and V consumption is a proxy of unhealthy diets that starts in childhood. This is a problem in Scotland where just 15% of children reported meeting the 5-a-day recommendation, and 10% did not consume any F and V on the previous day [8]. Furthermore, vegetables are less likely to be consumed compared with fruit as shown in the UK National Diet and Nutrition survey where 4 to 10-year old children ate 86 g of vegetables compared with 109 g of total fruit excluding juice [9]. Given that fussy eating is associated with low vegetable consumption, particularly bitter green vegetables [1], designing interventions to address this issue is important for child health.

Vegetable intake in young children is a complex multifactorial process and epidemiological, laboratory and observational studies have determined a number of developmental and environmental factors of importance. Whilst there is an innate dislike for bitter tastes, this can be overcome. Genetics may have an impact, but exposure in utero and during breast feeding is important to increase familiarity of bitter tastes [10]. The timing, frequency and variety of exposure at weaning with continuation through the early years are critical factors. Johnson (2016) suggests that parental influences are equally as important; crucially, parents have a major influence as role models, but other factors such as availability of vegetables in the home environment, parental intake and feeding styles may have an impact. In addition, socioeconomic influences such as parental income, maternal education and nutrition knowledge are consistently shown to be predictors of intake [11].

Repeated exposure is an effective method for increasing consumption of a target vegetable in preschool aged children [12,13,14]. Repeated exposure over 8–10 days increased acceptability of vegetables in infants and toddlers [15]. Repeated exposure has also had a positive effect on intake of a novel vegetable [16] and on increasing intake and liking for previously disliked vegetables [17,18]. Other approaches to encourage consumption of vegetables have used non taste sensory learning [19,20] to familiarise children to texture and smell while avoiding the pressure of having to taste them which might result in reduced consumption [21]. A sensory education programme, which included taste elements, was found to reduce food neophobia in older children aged 8–12 years [22]. However, there is limited evidence on the effectiveness of non taste sensory learning in younger children.

Child involvement in meal preparation has previously been associated with a higher preference for vegetables [23] and an increased willingness to try unfamiliar food items [24]. Cooking programmes involving parents and school aged children have shown small but sustainable improvements in F and V intake in families [25,26], but how this impacts children’s fussy eating behaviours in early years is not well established. The aim of this study was to evaluate the effect of a cooking skills programme (The Big Chef Little Chef (BCLC) programme) with the tandem participation of parents/carers and their 3–5-year old children on child food fussiness and willingness to try green vegetables. 

## 2. Materials and Methods 

### 2.1. Study Design and Ethics 

The evaluation had a quasi-experimental design. The study consisted of an intervention group who attended the BCLC programme and a comparison group recruited after the intervention from a similar population. This study was conducted in accordance with the guidelines laid down in the Declaration of Helsinki, and all procedures involving human subjects were approved by the Medical, Veterinary and Life Sciences (MVLS) Ethics Committee (Ref number 200170024).

### 2.2. Recruitment 

Invitation letters were sent to 94 council-based nurseries that participated in the High Five for Fruit Programme. This was a project ran by the registered charity Lanarkshire Community Food and Health Partnership and funded by North Lanarkshire Council, Scotland. High Five for Fruit aimed to increase F and V intake and promote healthy eating in families of preschool aged children (3–5 years) who attended nurseries in North Lanarkshire. The core activities of High Five for Fruit were free delivery of three portions of F and V per child each week and nutrition and health promotion via interactive sessions delivered by a nutritionist. Nine nurseries in North Lanarkshire contacted the High Five for Fruit nutritionist to register an interest in BCLC. These nurseries were used for both intervention and comparison recruitment. Participants were recruited using posters in the nurseries, targeting parents or carers who perceived their child to be a fussy eater. Nursery staff also referred children. The intervention group was a sample of convenience, as participation interest differed between nurseries. A similar number of parent/carer and child pairs were recruited for the comparison group.

Participants in the BCLC sessions were recruited and sessions delivered on site at the interested nine nurseries between October 2017 and May 2018. The comparison group was recruited in Summer 2018 and Spring 2019. Data were collected from intervention participants at nine council-based nurseries; however, one nursery was excluded from the data analysis as no comparison participants could be recruited.

To reduce contamination between the intervention and comparison group, all participants in the comparison group were recruited after the BCLC programme had taken place, and the inclusion criterion was that they had not taken part in BCLC. Participants in the comparison group received a high street voucher worth 10 GBP incentive if they attended both sessions, the incentive was given at the end of the tasting party. Intervention participants did not receive an incentive, but the 4 week BCLC was cost free, and they consumed foods or took ingredients home during each session.

**Figure 1:**
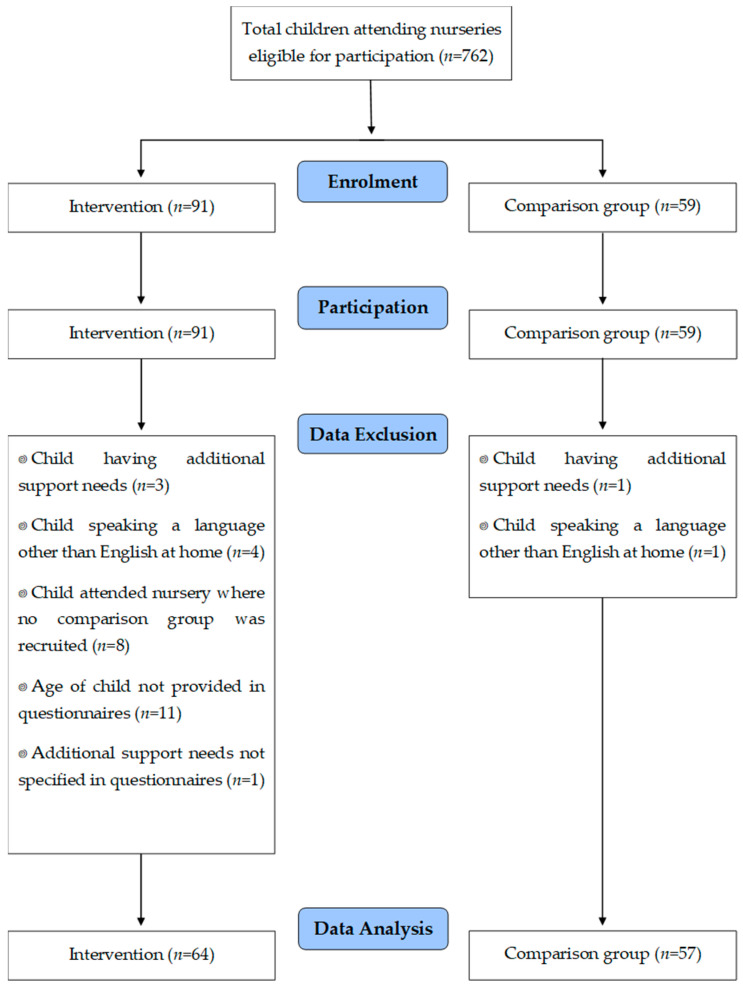
Participant recruitment and reasons for exclusion in data analysis. BCLC—Big Chef Little Chef.

The exclusion criterion (applied just to data analysis) was children who had additional support needs. In the UK, additional support refers to when more—or different—support to what is generally provided in educational establishments to children or young people of the same age is required. Many circumstances may mean that a child or young person needs additional support for learning e.g., disability or health, learning environment, family circumstances, social and emotional factors. These children were excluded from data analysis because their eating behaviours might be different from other children. Children with different ethnic backgrounds defined by speaking a language other than English or Gaelic at home were also excluded from data analysis as their dietary patterns for fruit and vegetable consumption might be influenced by cultural and ethnical exposure that is different to Scottish/British households. Data were collected from 142 parent/carer and child dyads, but complete data were available for 121 pairs (Figure 1).

### 2.3. The BCLC Programme Intervention and Comparison Group

BCLC for fussy eaters was developed because of parental and nursery staff concerns about fussy eating behaviours among children attending North Lanarkshire nurseries. It consisted of interactive sessions with parent/carers and their nursery aged child for 1.5 h once a week over four weeks (a total of 6 h for parents and children). Parents and children prepared a meal and snack each week in a relaxed and playful environment. Children were encouraged to play with cooking utensils for the development of simple food preparation skills such as using a knife, grating, mixing and measuring ingredients. Repeated exposure and sensory learning were incorporated in each session. For repeated exposure, the children worked with a wide variety of vegetables and herbs and were gently encouraged to try ingredients together with their parent. Several vegetables were used on multiple occasions over the 4 week course and particularly those that were less likely to be consumed at home such as peppers (red, yellow and green), spinach, butternut squash and tomatoes. BCLC also incorporated sensory learning focusing on sight, smell, touch and taste. In week 1, for example, colour was highlighted; children were encouraged to name and try all the different colours of vegetables used for their pizzas and rainbows. In week 2, the focus was on touch and texture, particularly the transformation of couscous from hard and crunchy to warm, soft and fluffy with the addition of stock. Flavour was explored in week 3, when children were asked to compare different tastes including dried cranberries, spinach, butternut squash, celery, cream cheese, olive oil and balsamic vinegar. Smell was encouraged at each weekly session, for example when rubbing garlic and sprinkling oregano onto pizzas, tearing up fresh herbs and using foods with strong smells such as tuna. Children were given an extra sticker at the end of each session if they had tried something new that day. The activities delivered weekly are shown in Table 1. The programme was adapted from the ongoing health promotion activities of High Five for Fruit which represents an intervention in real life settings.

A “green vegetable tasting party” was delivered at the last session of the programme (week 4). This allowed children to taste bite-sized amounts of the following green vegetables, both raw and cooked: broccoli, cabbage, green beans, sugar snap peas, spinach and green pepper. Green vegetables were chosen as children typically dislike them [27]. Both raw and cooked vegetables were provided as previous evidence has suggested there is a differing preference between them [28,29]. All vegetables were prepared prior to the session; the cooked vegetables were steamed with no seasoning, refrigerated and were given to the children cold.

Participants in the comparison group were invited to attend two sessions. The first session at week 1 was a parent/carer talk on healthy eating focusing on Eatwell Guide and reducing free sugar consumption which are part of the standard health promotion activities delivered by the High Five for Fruit programme. For the purpose of this study, tips for fussy eaters were also included. The session lasted 1 hr. The second session took place three weeks after (week 4) and lasted 0.5 h. The total time of contact with the comparison group was 1.5 h for parents/carers and 0.5 h for children. For this second session, participants (dyads) were invited to a “green vegetable tasting party” identical to that in the final week of BCLC. The participants completed the same pre and post questionnaires as the intervention group.

### 2.4. Outcome Measurements, Sample Size and Evaluation Methodology

The primary outcome was food fussiness score measured with a questionnaire. The secondary outcome was willingness to try vegetables at the tasting party.

Sample size was calculated posthoc (Altman, 1980) based on a reduction of 0.5 points in the food fussiness score between comparison and intervention, where alpha  =  0.05, power  =  0.80. This found that a sample of *n* = 130 participants was required in total and *n* = 65 per group for the main outcome measure (food fussiness score).

For food fussiness, we used six statements from the previously validated Children’s Eating Behaviour Questionnaire (CEBQ) [30]. This six-item list was validated in 752 children (mean age 6.7 years) against a psychiatric interview that determined if the child had “normal”, “moderate” or “severe” pickiness [31]. The scale was found to effectively categorise children when using cut-offs based on sensitivity and specificity, with a score of 3.33 for “severe” and 3.00 for “moderate” cases of pickiness. In our study, we modified the answers to facilitate participants responses as the scale was used prior to this evaluation and parents expressed difficulties understanding it. We used scales from 1 to 5 for “completely disagree” to “completely agree”. Each statement was scored depending on the direction from 1 (“completely disagree”) to 5 (“completely agree”) or 5 (“completely disagree”) to 1 (“completely agree”) as per the CEBQ scoring system. An average score was calculated using the scores for each statement added together and divided by six. Final scores ranged from one (lowest food fussiness) to five (highest food fussiness). If any statements were unanswered, a score was not calculated, and this was entered as missing data in IBM SPSS (Statistics for Windows Version 24.0. IBM Corp., Armonk, NY, USA).

Tasting party scores were calculated for each vegetable, both raw and cooked. A score between one and three was given depending on if the vegetable was tried or eaten, with 1 = “did not try”; 2 = “tried it/ate some”; 3 = “ate it all”. An average score for raw and cooked vegetables was calculated.

Questionnaires for food fussiness were completed before (week 1) and after (week 4) for both intervention and comparison group. The tasting scores were measured at one point at the end of the study period (week 4).

### 2.5. Data and Statistical Analysis

All data were analysed using IBM SPSS statistical software version 24.0. Normality was tested for all data using the Shapiro–Wilk test. Descriptive statistics described demographic data using parametric or nonparametric data as required. To test differences between intervention and comparison groups for primary and secondary outcomes, an independent-samples T-test was used for parametric data, while a paired-sample T-test was used to determine differences within groups between week 1 and week 4. For non-normally distributed data, a two-sample Mann–Whitney test was used in place of an independent-samples T-test, and a Wilcoxon signed-rank test was used in place of a paired-sample T-test. Data were missing from the primary and outcome variables in 32.4% of cases, with 19.9% of all values missing. Little’s Missing Completely at Random test on the full dataset was significant (Chi-squared (101) = 223.9, *p* < 0.01) indicating that the data were not missing completely at random, and hence a complete-case analysis with listwise deletion would not be an acceptable strategy to deal with the missing values. Missing data were imputed using a multiple imputation method with fully conditional specification and 10 iterations, generating 50 complete datasets. For imputed data analysis, parametric test statistics were pooled using the standard functions of SPSS; for nonparametric tests, the standardised test scores were combined using Rubin’s Rules [32] to produce a t-value and associated degrees of freedom, from which a *p*-value was calculated. Results were considered significant at the *p* < 0.05 level.

## 3. Results

Data were collected from 142 parent/carer and child dyads, but data were analysed for 121 pairs (Figure 1). The number of participants and demographic characteristics are shown in Table 2. No statistical differences were observed for any of the demographic variable. For the BCLC intervention, 69% participants attended all sessions and a further 31% attended 3 sessions.

### 3.1. Food Fussiness

**Figure 2:**
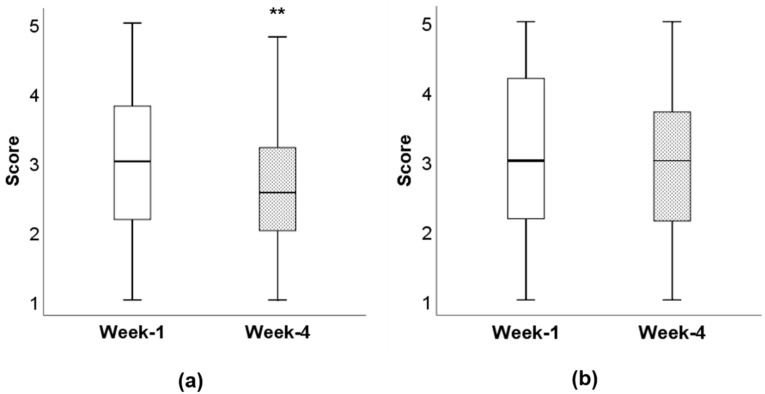
Food fussiness scores at week 1 and time week 4: (**a**) food fussiness scores in the intervention group at week 1 and week 4, ** *p* = 0.002. (**b**) Food fussiness scores in the comparison group at week 1 and week 4, *p* = 0.400. Scores are an average of six items; scores ranged from 1 to 5 for “completely disagree” to “completely agree”.

The intervention group food fussiness score significantly decreased between week 1 (3.0) and week 4 (2.6) (*p* < 0.01), while no significant change was seen between time points (week 1 and week 4) in the comparison group (3.1 and 3.0, respectively) (Figure 2). The scores remained statistically significant for the intervention group in the imputed data analysis (z = −2.5433, *p* = 0.011), while no significant differences were observed in the comparison group (z = −0.69664, *p* = 0.486). The median difference between week 1 and week 4 for the intervention group was 0.346 (P25 -0.01; P75 0.807) and 0.133 (P25 -0.223; P75 0.600) for the comparison group without statistical significance between intervention and comparison groups.

### 3.2. Willingness to Try Vegetables

**Figure 3:**
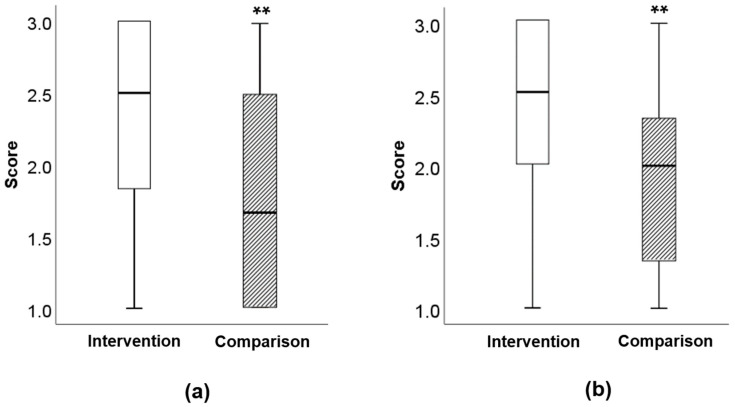
Willingness to try vegetables at tasting party. (**a**) Taste scores for cooked vegetables, ** *p* < 0.001. (**b**) Taste scores for raw vegetables, *p* < 0.001. Scores are an average of six vegetables; scores ranged from 1 = “did not try”; 2 = “tried it/ate some”; 3 = “ate it all”.

Children who participated in BCLC had significantly higher average tasting party scores compared with the comparison group, for both raw and cooked vegetables (both *p* < 0.001, Figure 3). No differences were found between average raw and cooked vegetable scores in the intervention group; however, the average cooked vegetable score was significantly lower (*p* < 0.05) than the average raw score for the comparison group. The intervention group also had significantly higher individual vegetable scores for all vegetables (Table 3). In both groups, children had significantly lower scores for cooked spinach compared with raw spinach. There were no differences between raw and cooked vegetables for any other individual vegetables. The tasting party scores did not change in the complete case analysis when compared with the imputation data analysis scores, except for one variable (raw broccoli) (Supplementary Table S1).

## 4. Discussion

The BCLC programme was successful in reducing the food fussiness score between time points in children who participated in the intervention. The effect size for participants in the intervention group was small (~0.3), while no change was observed between time points in the comparison group. Nevertheless, this small change meant that children who were classified initially as “moderate” cases of pickiness [31] in the intervention group moved to not being picky at the end of the 4 week programme, while there was no change in the comparison group. Considering that the measurements for pickiness are based on parental perception, one cannot rule out subjectivity that might be based on increased neophobic responses to new foods that are common in this age group [6].

Our programme was constructed based on a multicomponent approach that could have had an impact on the small but significant reduction in food fussiness. Children in the programme were exposed to vegetables via repeated exposure, this could have reduced their neophobic responses and been perceived by the mother as reduced fussiness instead. In food neophobic children, refusal of unfamiliar foods is typically reduced after 15 exposures, while it is not reduced in children who are fussy [1]; however, in our study, the number of exposures was lower due to the duration of the programme. Several vegetables were offered multiple times over the 4-week BCLC programme and parents were given information on the importance of repeated exposure as part of the intervention. Nevertheless, we did not measure exposure times in detail as this was a free living, less intense, but more realistic intervention. We considered this approach to be more sustainable and applicable to real life settings than a more controlled intervention in a study setting. The sensory learning element could have also contributed to familiarisation with vegetables, and in turn had an effect on the food fussiness score. Children (age 7–9 years) who prepared and cooked food during an intervention had a reduction in food neophobia and fussiness score, although this was not significant [33]. As the children in this study were older than those in the current study, there is potential that cooking skills may have a stronger effect on fussiness in younger children. The programme may have relieved some parent or caregiver’s worries about their child’s eating behaviours after viewing them in a different setting, where they were perhaps more willing to try new or previously disliked foods. This may have led to them viewing their child as less fussy when filling out the questionnaire at week 4.

Children in the intervention group had significantly higher overall scores for trying both raw and cooked vegetables, compared with the comparison group. They also had significantly higher scores for all individual raw and cooked vegetables. There was a lower score in both groups for the cooked spinach, potentially due to its appearance and texture when steamed. A qualitative study found that children described the negative aspects of vegetables, particularly cooked, as “mushy”, “squishy” “smooshy” and “cold” [34]. A study where vegetables were sautéed in garlic and onion seasoning found children preferred cauliflower, bell peppers, green beans and celery cooked instead of raw, although only cauliflower reached statistical significance [35]. However, there is still potential that parents may have under or over reported their child’s willingness to try. Furthermore, a major limitation of this secondary outcome was the lack of baseline data as the taste party took place just at the end of the study.

The exposure to certain vegetables during BCLC might have caused the increased willingness to try. Previous evidence shows that providing children with a choice of two or more vegetables can increase intake of vegetables [36,37,38]. This may have led to some children being more willing to try or eat the vegetables during the tasting party as each child was given a plate with all six vegetables on it together. Nonfood rewards, such as stickers, have been found to successfully increase liking and intake of a previously disliked vegetable when coupled with repeated exposure [17]. Stickers were used at the end of sessions for participation and encouragement. The sensory learning elements of the programme may also have had an effect, as previous sensory learning interventions have been successful in increasing children’s willingness to taste vegetables [19,20]. This may have been through familiarising the children with the different vegetables used in the programme. The non taste sensory elements of the programme may have been particularly useful, as this avoids the pressure of tasting, which has been found to have a negative effect on eating behaviours [21]. The involvement in cooking during the programme may also have encouraged the children to try more vegetables, having previously been associated with an increased intake of salad [26] and an increased willingness to try foods that are unfamiliar to the child [24]. There is potential that peer influence may have resulted in some children trying or eating more vegetables than they wanted to. Children have previously been found to pick a nonpreferred vegetable over a preferred vegetable if peers with opposite food preferences are seated with them [39].

A further explanation of the positive findings of this study is the longer duration of interactions between children, parents and the nutritionist in the intervention group compared with the comparison group (6 h vs. 1.5 h, respectively, for parents/carers and 6 h vs. 0.5 h, respectively, for children). Children could have become more responsive to the nutritionist and also social desirability bias towards the researcher could not be ruled out.

### Strengths and Limitations

Our study objectively measured children’s willingness to try vegetables, which is a key stage before improving consumption of vegetables [11], as well as measuring actual consumption of vegetables during a tasting party, and it is a strength that we considered both aspects. Additionally, a strength of the tasting party was that the cooked vegetables were not seasoned, which reduced the likelihood of children only trying the vegetable for the seasoning, rather than for the taste of the vegetable. It was beneficial to provide the children with both raw and cooked vegetables to see if there were any differences between willingness to try. The cooked vegetables were given to the children cold. This may have affected the willingness to try these vegetables, as a previous study found that children described one of the negative aspects of cooked vegetables as “cold” [34]. This was conducted in both groups, and the children in the intervention group were still significantly more willing to try the cooked vegetables compared with the comparison group, showing that the intervention had a positive effect.

A strength of this study was the comparison group, although we used a quasi-experimental design, this allowed us to compare intervention effects. There were no significant differences between the number of participants in the groups or between the demographic characteristics of the groups, which suggest no selection bias. A further strength is that the BCLC programme was carried out in a real-life setting within nurseries, showing that there is potential to make changes to children’s eating behaviours during their education time. Even though BCLC was mainly focused on improving the children’s diets and eating behaviours, it was important to have the parents and carers also attend. Parental influence through role modeling and providing access to F and V are both key factors in child F and V consumption [34].

A limitation was the modification of the validated CEBQ statements for measuring food fussiness score, this was conducted because parents in similar cohorts in previous sessions delivered prior to BCLC found it confusing.

Although BCLC aimed to reduce fussy eating in participating children, the course content was heavily focused on exposing children to vegetables. This does have benefits, as fussy eating in children is associated with a reduced intake of vegetables [4,5]. This may have only helped children who were fussy, or perhaps neophobic, in regard to vegetables and not benefited the children with more severe fussiness. It is also possible that for severe food fussiness, more work would be required than a four-week programme. Even for moderate fussiness, four weeks may not be enough to establish long-term changes. There were no follow-up questionnaires for those who participated in the study, so it is not clear if longer-term effects have occurred and whether the programme has a sustained effect.

Participants in the comparison group received a small monetary incentive at the end of the tasting party, this was not given to the intervention group who instead consumed meals or took them home. We cannot account for the incentive effect in this study.

We acknowledge that the intake of vegetables in young children is multifactorial, and here we have considered one factor in isolation. Whilst this is a limitation, it is beyond the scope of this research to consider all factors that influence intake.

## 5. Conclusions

The nursery-based BCLC programme significantly reduced food fussiness score, and children in the intervention group had a significantly increased willingness to try both raw and cooked green vegetables than those in the comparison group. This was achieved through incorporating techniques previously shown to increase F and V consumption in children of this age group. Although further work is required, this shows there is potential for cooking skills programmes such as BCLC to have positive effects on preschool children’s diet and eating behaviours.

## Figures and Tables

**Table 1:** BCLC Weekly Activities.

	Take Home Dish	Snack	Vegetable Exposure	Information Provided to Parents and Rewards for Children
**Week 1** **Colour: Eat a Rainbow!**	Pitta bread pizzas	Vegetable rainbow with rainbow dips	Cherry tomatoes, red/green/yellow pepper, sweetcorn, olives, oregano, garlic, red cabbage, broccoli, butternut squash, spinach.	Fussy eating strategies Stickers for tasting new foods
**Week 2** **Texture**	Vegetable couscous	Vegetable butterflies	Red/green/yellow pepper, cherry tomatoes, cucumber, spring onions, sweetcorn, mint.	Stickers for tasting new foods
**Week 3 Flavour**	Butternut squash and cranberry pasta salad	Vegetable snails/dragonflies	Butternut squash, spinach, cucumber, celery, red/green/yellow peppers, peas, chives.	Stickers for tasting new foods
**Week 4** **Greens: Cooked vs. Raw**	Tuna rainbow wrap	Fruit car	Spinach, red/green/yellow pepper, carrot. During tasting party: broccoli, cabbage, green beans, sugar snap peas, spinach and green pepper.	Stickers for tasting new foods

**Table 2:** Demographic characteristics of intervention and comparison participants and children.

	Intervention		Comparison	
	*n*	%	*n*	%
Participants	64	52.9	57	47.1
Type of Participant				
Parent	49	76.7	49	86.0
Grandparent	9	14.1	7	12.2
Carer	1	1.6	0	0.0
Missing data	5	7.8	1	1.8
Sex				
Female	57	89.1	53	93.0
Male	5	7.8	3	5.2
Missing data	2	3.1	1	1.8
Age (years)				
17–24	3	4.7	3	5.3
25–34	12	18.8	21	36.8
35–44	20	31.3	18	31.6
≥45	10	15.6	13	22.8
Missing data	19	29.3	2	3.5
Highest Education Level				
No Formal Qualifications	4	6.3	3	5.3
Scottish Standard Grades or GCSEs	10	15.6	17	29.8
Scottish Highers or ‘A’ Levels	9	14.1	8	14.0
Degree (or equivalent)	19	29.7	20	35.1
Other *	2	3.1	6	10.5
Missing data	20	31.3	3	5.3
Socioeconomic Index of Deprivation ^$^				
Quintile 1	7	10.9	10	17.5
Quintile 2	14	21.9	17	29.8
Quintile 3	16	25.0	13	22.8
Quintile 4	5	7.8	7	12.3
Quintile 5	2	3.1	0	0.0
Missing data	20	31.3	10	17.5
**Children**				
Sex				
Female	36	56.3	33	57.9
Male	28	43.8	24	42.1
Age (months)				
36–42	18	28.1	21	36.8
43–49	16	25.0	20	35.1
50–56	24	37.5	9	15.8
57–64	6	9.4	7	12.3

* For education, “other” included Scottish Vocational Qualification Level 3, City & Guilds (no level given), National Vocational Qualification (no level given), Higher National Certificate and Higher National Diploma. ^$^ Calculated using the Scottish Index of Multiple Deprivation classification, Quintile 1 represents the most deprived household and Quintile 5 the most affluent. GCSE: General Certificate of Secondary Education.

**Table 3:** Individual vegetable scores from green vegetable tasting party for intervention and comparison group.

Tested Vegetable	Intervention		Comparison	
	Median	P25, P75	Median	P25, P75
Raw				
Broccoli	3.0	2.0, 3.0	2.0 *	2.0, 3.0
Cabbage	3.0	2.0, 3.0	2.0 *	2.0, 3.0
Green Beans	3.0	2.0, 3.0	2.0 ***	1.0, 2.0
Spinach	3.0	2.0, 3.0	2.0 **	1.0, 3.0
Green Pepper	3.0	2.0, 3.0	2.0 ***	1.0, 2.0
Sugar Snap Peas	3.0	2.0, 3.0	2.0 **	1.0, 3.0
Cooked				
Broccoli	3.0	2.0, 3.0	2.0 **	1.0, 3.0
Cabbage	3.0	2.0, 3.0	2.0 *	1.0, 3.0
Green Beans	3.0	2.0, 3.0	2.0 *	1.0, 3.0
Spinach	2.0^†^	1.0, 3.0	1.0 ***^††^	1.0, 2.0
Green Pepper	3.0	2.0, 3.0	1.0 ***	1.0, 2.0
Sugar Snap Peas	3.0	2.0, 3.0	2.0 **^†^	1.0, 2.0

* Difference between groups (* *p* < 0.05; ** *p* < 0.01; *** *p <* 001) by a two-sample Mann–Whitney test. ^†^ Difference between raw and cooked vegetables (^†^ *p* < 0.05; ^††^ *p* < 0.01) found using a Wilcoxon signed-rank test. Scores ranged from 1 = “did not try”; 2 = “tried it/ate some”; 3 = “ate it all”.

## Supplementary Materials

The following are available online at https://www.mdpi.com/2072-6643/12/9/2623/s1, Table S1: Vegetable scores from vegetable tasting party for intervention and comparison groups using completed data and imputed data.
