# Supplementary material for: A Nursery-Based Cooking Skills Programme with Parents and Children Reduced Food Fussiness and Increased Willingness to Try Vegetables: A Quasi-Experimental Study
Source: Nutrients. 2020 Aug 28;12(9):2623. doi: 10.3390/nu12092623 (PMC7551038; doi:10.3390/nu12092623)
Supplement: Supplementary file 1 [file nutrients-12-02623-s001.pdf]

Supplementary Table 1. Vegetable scores from vegetable tasting party for intervention and comparison groups using completed data and imputed data

Tasted Vegetable	Completers		Imputation	
	Z score	P value	Z score	P value
Total Raw Score	-3.72	<0.001	-3.59	<0.001
Raw Broccoli	-1.98	0.048	-1.68	0.094
Raw Cabbage	-2.53	0.011	-2.39	0.018
Raw Green Beans	-3.91	<0.001	-3.60	<0.001
Raw Spinach	-3.31	0.001	-3.11	0.002
Raw Green Pepper	-4.51	<0.001	-4.06	<0.001
Raw Sugar Snap Peas	-2.91	0.004	-2.743	0.007
Total Cooked Score	-3.83	<0.001	-3.67	<0.001
Cooked Broccoli	-3.04	0.002	-2.95	0.004
Cooked Cabbage	-2.44	0.015	-2.38	0.019
Cooked Green Beans	-2.35	0.019	-2.22	0.028
Cooked Spinach	-3.56	<0.001	-3.27	0.001
Cooked Green Pepper	-4.29	<0.001	-3.97	<0.001
Cooked Sugar Snap Peas	-3.38	0.001	-3.10	0.002

Total scores are an average of six vegetables either raw or cooked: Scores ranged from 1-3, 1 = 'did not try'; 2 = 'tried it/ate some'; 3 ='ate it all'.
